# Non-uniform mixing of hepatic venous flow and inferior vena cava flow in the Fontan conduit

**DOI:** 10.1098/rsif.2020.1027

**Published:** 2021-04-07

**Authors:** Friso M. Rijnberg, Séline F. S. van der Woude, Hans C. van Assen, Joe F. Juffermans, Mark G. Hazekamp, Monique R. M. Jongbloed, Sasa Kenjeres, Hildo J. Lamb, Jos J. M. Westenberg, Jolanda J. Wentzel, Arno A. W. Roest

**Affiliations:** ^1^ Department of Cardiothoracic Surgery, Leiden University Medical Center, Leiden, The Netherlands; ^2^ Department of Radiology, Leiden University Medical Center, Leiden, The Netherlands; ^3^ Department of Cardiology and Anatomy and Embryology, Leiden University Medical Center, Leiden, The Netherlands; ^4^ Department of Pediatric Cardiology, Leiden University Medical Center, Leiden, The Netherlands; ^5^ Department of Cardiology, Biomedical Engineering, Erasmus MC, Rotterdam, The Netherlands; ^6^ Department of Chemical Engineering, Faculty of Applied Sciences, Delft University of Technology and J. M. Burgerscentrum Research School for Fluid Mechanics, Delft, The Netherlands

**Keywords:** hepatic flow distribution, flow, Fontan, hepatic venous, mixing, computational fluid dynamics

## Abstract

Fontan patients require a balanced hepatic blood flow distribution (HFD) to prevent pulmonary arteriovenous malformations. Currently, HFD is quantified by tracking Fontan conduit flow, assuming hepatic venous (HV) flow to be uniformly distributed within the Fontan conduit. However, this assumption may be unvalid leading to inaccuracies in HFD quantification with potential clinical impact. The aim of this study was to (i) assess the mixing of HV flow and inferior vena caval (IVC) flow within the Fontan conduit and (ii) quantify HFD by directly tracking HV flow and quantitatively comparing results with the conventional approach. Patient-specific, time-resolved computational fluid dynamic models of 15 total cavopulmonary connections were generated, including the HV and subhepatic IVC. Mixing of HV and IVC flow, on a scale between 0 (no mixing) and 1 (perfect mixing), was assessed at the caudal and cranial Fontan conduit. HFD was quantified by tracking particles from the caudal (HFD_caudal conduit_) and cranial (HFD_cranial conduit_) conduit and from the hepatic veins (HFD_HV_). HV flow was non-uniformly distributed at both the caudal (mean mixing 0.66 ± 0.13) and cranial (mean 0.79 ± 0.11) level within the Fontan conduit. On a cohort level, differences in HFD between methods were significant but small; HFD_HV_ (51.0 ± 20.6%) versus HFD_caudal conduit_ (48.2 ± 21.9%, *p* = 0.033) or HFD_cranial conduit_ (48.0 ± 21.9%, *p* = 0.044). However, individual absolute differences of 8.2–14.9% in HFD were observed in 4/15 patients. HV flow is non-uniformly distributed within the Fontan conduit. Substantial individual inaccuracies in HFD quantification were observed in a subset of patients with potential clinical impact.

## 1. Introduction

The Fontan operation is the palliative procedure for single-ventricle patients, in which both venae cavae are connected with the pulmonary arteries (PAs), also called the total cavopulmonary connection (TCPC). The TCPC needs to ensure a balanced hepatic venous flow distribution (HFD), containing an important hepatic factor, towards both lungs [1]. A lack of hepatic factor has been associated with the formation of pulmonary arteriovenous malformations in the affected lung, leading to progressive hypoxaemia, cyanosis and exercise intolerance [2].

In recent years, patient-specific platforms using a combination of computational fluid dynamics (CFD) and magnetic resonance imaging (MRI) are emerging as a valuable tool for clinicians for evaluating HFD and power loss [3,4]. In addition, these platforms allow for performing ‘virtual surgery', by which multiple different TCPC geometries can be virtually created and associated blood flow can be predicted. Subsequently, the optimal TCPC geometry with minimal power loss and a balanced HFD can be determined thereby guiding surgical and catheter-based interventions [5,6].

HFD can be determined with particle tracing techniques using four-dimensional flow MRI [7–11] or CFD models [12]. Conventional HFD quantification methods track particles that are uniformly seeded within the Fontan conduit and determine the distribution of these particles towards both PAs. Therefore, this method relies on the unvalidated assumption that hepatic blood is uniformly distributed within the Fontan conduit [13,14]. However, since blood flow is laminar in the inferior vena cava (IVC) and Fontan conduit, the mixing of hepatic blood with IVC blood might be less optimal than generally assumed. We hypothesized that there is a non-uniform distribution of hepatic venous flow in the Fontan conduit that affects the accuracy of the current HFD quantification approach. These inaccuracies have potential consequences for the identification of patients with unbalanced HFD, or by affecting optimal TCPC model selection in virtual surgery platforms. The aim of this study was twofold: (i) to test the hypothesis of non-uniform hepatic blood distribution within the Fontan conduit by quantification of mixing between IVC and hepatic venous flow within the Fontan conduit and (ii) to quantify HFD from the level of the Fontan conduit (conventional method), as well as from the level of the hepatic veins (HVs, direct method).

## 2. Methods

### 2.1. Patient population

Fifteen Fontan patients were included that underwent MRI as part of a prospective study between November 2018 and May 2019 at the Leiden University Medical Center. All patients greater than 8 years old without contraindications for MRI were eligible for inclusion. Patient characteristics are provided in table 1. The study was approved by the institutional review board of the hospital. Informed consent was obtained from all subjects and/or their guardians.

**Table 1.** Patient characteristics.

male/female	9/6
BSA (m^2^)	1.6 (0.2)
age at MRI (years)	18.2 (5.6)
Fontan type (ECC/LT)	14/1
conduit size (16/18/20 millimetre)	9/4/1
Q_IVC_ (l min^−1^)	3.0 (0.7)
Q_HV_ (l min^−1^)	1.5 (0.6)
contribution of *Q*_HV_ to *Q*_conduit_ (%)	32.7 (9.3)

Values are reported as mean (standard deviation). BSA, body surface area (Haycock); MRI, magnetic resonance imaging; ECC, extracardiac conduit; LT, lateral tunnel; Q, flowrate; IVC, inferior vena cava; HV, hepatic veins.

### 2.2. Magnetic resonance imaging

MRI acquisition details are provided in electronic supplementary material, table S1. Transversal and sagittal stacks of static, respiratory-compensated two-dimensional anatomic images were acquired for segmentation of the TCPC. Free-breathing, two-dimensional phase-contrast MRI (2D PC-MRI) with three-directional velocity encoding was acquired at the following locations: subhepatic IVC, Fontan conduit, superior vena cava (SVC), right (RPA) and left PAs (LPA). In this study, the flow was quantified using the (clinically standard) through-plane velocity direction only (CAAS MR Solutions v. 5.1, Pie Medical Imaging, Maastricht, The Netherlands). The total hepatic venous flow was determined by subtracting IVC flow from Fontan conduit flow.

### 2.3. Three-dimensional total cavopulmonary connection model creation

A detailed description of the TCPC segmentation and CFD analysis are provided in electronic supplementary material 1. The TCPC was segmented using both sagittal and transversal stacks, covering the area between the subhepatic IVC, hepatic veins, SVC, RPA and LPA (ITK-SNAP [15]). Segmental branches were excluded, except for the right upper lobe branches. A three-dimensional TCPC model was created, smoothed and centrelines were derived for each vessel (VMTK [16]).

### 2.4. Computational fluid dynamics simulations

All inlets and outlets of the three-dimensional TCPC model were clipped perpendicular to the centrelines. Furthermore, vessel extensions were added to the entrances and exits of the model. Using these vessel extensions, the inflow velocity profiles at the entrances of the three-dimensional TCPC model were fully developed and resembled the velocity profiles at those locations. After adding vessel extensions at all inlets and outlets, the three-dimensional TCPC models were meshed with 30 polyhedral elements across the average vessel diameter (range 0.4–0.5 mm elements) in order to achieve mesh-independent results (ANSYS ICEM v. 17.1, Inc., Canonsburg, PA) [17]. All CFD simulations were performed using commercially available Fluent software (v. 17.1, ANSYS, Inc., Canonsburg, PA).

Time-resolved flowrates were prescribed with a parabolic velocity profile at the inlets. The total hepatic venous flow was divided over the multiple hepatic veins based on the ratio of their respective cross-sectional areas. Outlet boundary conditions were imposed based on the ratio of measured pulmonary flow distribution (RPA/LPA flow divided by the total PA flow) [3]. A rigid vessel wall was assumed and a no-slip condition prescribed. Blood flow was assumed to be laminar. A Carreau model was used to account for the non-Newtonian blood properties in the TCPC [18].

### 2.5. Quantification of mixing between inferior vena caval and hepatic blood flow

A method to quantify the mixing of hepatic and IVC blood flow within the Fontan conduit was developed based on the spatial distribution of both flows within the Fontan conduit using particle tracing (Paraview.org). A total of 7500 particles were released from the hepatic veins for each time step during five cardiac cycles. The number of particles per hepatic vein was based on the ratio of their respective cross-sectional areas. The amount of particles released from the subhepatic IVC was based on the average flow ratio between the IVC and hepatic veins; $7500\times (Q_{\mathrm{IVC}}/Q_{\mathrm{HV}})$.

**Figure 1.**
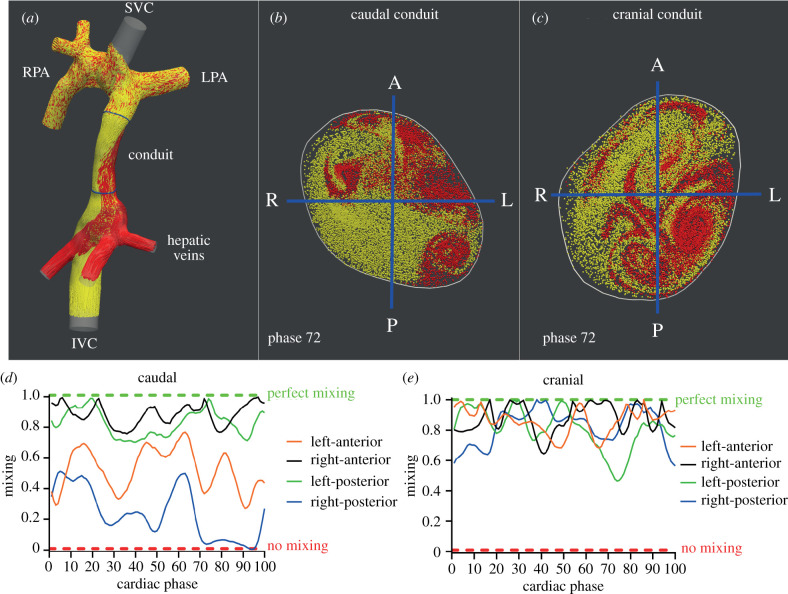
(*a*) Pathlines originating from the hepatic veins (HV, red) and inferior vena cava (IVC, yellow) are shown within the TCPC of a typical extracardiac conduit Fontan patient. Positions of the caudal and cranial cross-sections with the Fontan conduit are shown (blue). Of note, no pathlines were generated from the superior vena cava to allow for better visualization of the HV and IVC flow. (*b*,*c*) Transections with HV and IVC pathlines are shown for the caudal (*b*) and cranial (*c*) cross-sections for phase 72/100 of the cardiac cycle. The division into four subsections is indicated by blue lines. Note how an evidently non-uniform distribution of HV flow is present, most strikingly at the caudal part of the conduit, with almost no HV flow present in the right-posterior subsection. (*d*,*e*) Time-resolved mixing characteristics of HV and IVC flows are shown for the caudal and cranial cross-section of the conduit. At the caudal conduit, a relatively good mixing is present in the right-anterior and left-posterior subsections, with reduced mixing in the left-anterior and right-posterior segments. Note how almost no mixing is present at phase 72 in the right-posterior segment, consistent with the absence of HV flow in this subsection (*b*). (*e*) Significantly better mixing is present at the cranial part of the conduit, illustrated by a more uniform distribution of IVC and HV flow streams over the cross-section. SVC/IVC, superior/inferior vena cava; LPA/RPA, left/right pulmonary artery; A, anterior; P, posterior; L/R, left/right.

Particles were released from the HVs and IVC for each time step during five cardiac cycles. Pathlines, representing the trajectory each particle follows over time, were generated and transections with these pathlines were made at two cross-sections with the Fontan conduit: the caudal part of the Fontan conduit, just above the connection between the Fontan conduit with the IVC/HVs, and at the cranial part of the Fontan conduit, just below the connection with the PA (figure 1*a*–*c*; electronic supplementary material, video S1). The ratio of IVC and HV pathline transections at each cross-section of the Fontan conduit were recorded for each time step in the 5th cardiac cycle.

Subsequently, both caudal and cranial Fontan conduit cross-sections were manually subdivided in a right–left and anterior–posterior direction, resulting in four subsections: left-anterior, left-posterior, right-anterior and right-posterior (figure 1*b*,*c*). The ratio of IVC and HV pathline transections was determined in each of the four subsections. Next, mixing (M) in each of the four subsections was determined for each time-phase of the cardiac cycle by comparing the ratios of IVC and HV pathline transections within the entire cross-section with the ratios of IVC and HV pathline transections within each of the four subsections, ranging from 0 (no mixing) to 1 (perfect mixing). A mixing of 0 indicates no mixing (only HV or IVC pathlines present in the subsection) and 1 indicates perfect mixing (the exact same ratio of HV and IVC pathlines in the subsection as in the entire cross-section). Furthermore, cardiac cycle averaged mixing (*M*_average_) was determined for each subsection and for the entire cross-section. A detailed description of the mixing quantification method is provided in electronic supplementary material S2.

### 2.6. Hepatic blood flow distribution quantification

**Figure 2.**
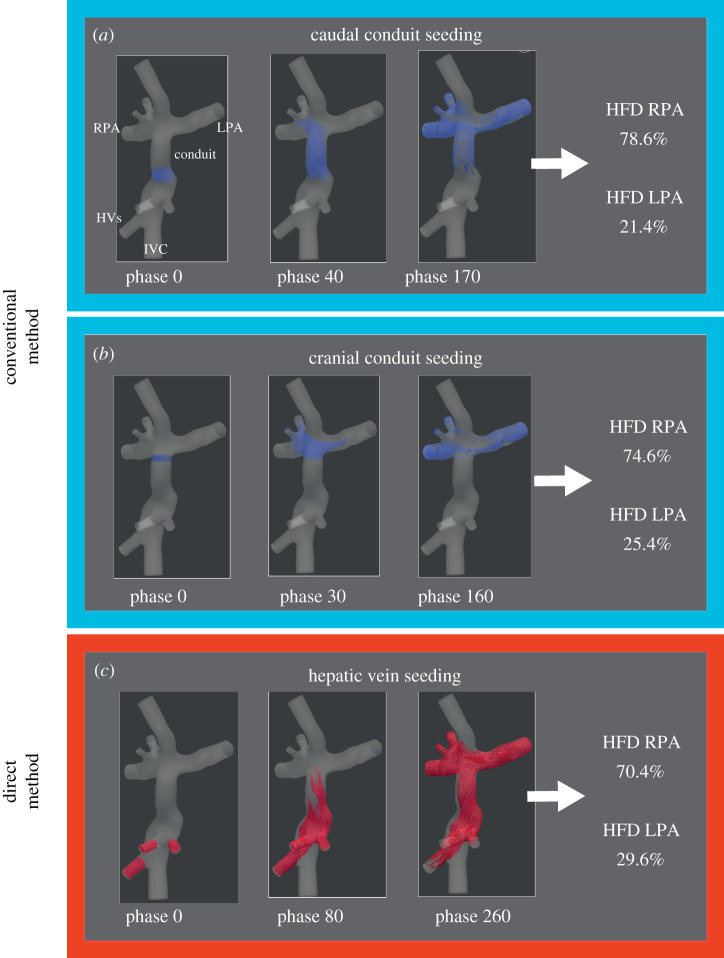
The three HFD quantification approaches are shown for a typical Fontan patient. Particles were uniformly seeded from the caudal or cranial conduit (conventional method) or directly from the hepatic veins (direct method). The starting position and the trajectory of these particles over time are shown for two cardiac phases. The percentage of particles arriving at each pulmonary artery were recorded representing the HFD. IVC, inferior vena cava; HVs, hepatic veins; LPA/RPA, left/right pulmonary artery; HFD, hepatic flow distribution.

HFD was quantified using particle tracing [7,13,19], by seeding 7500 particles from either the HVs (HFD_HV_; direct method) or from the level of one of the two cross-sections (HFD_caudal conduit_ and HFD_cranial conduit_; conventional method, figure 2). The 7500 particles were divided over the hepatic veins based on the ratio of their respective areas. Particles were released for 100 time steps (1 cardiac cycle) and particles arriving at the PAs were recorded for 500–1200 time steps (5–12 cardiac cycles) to allow for sufficient transit time for particles to reach the PAs. HFD is defined as the ratio of particles exiting through the LPA with respect to the total number of particles reaching either PA [13]:

$$\mathrm{HFD}=\frac{P_{\mathrm{LPA}}}{P_{\mathrm{RPA}}+\;P_{\mathrm{LPA}}}\;\times \;100\text{\%},$$

where *p* is the number of particles arriving in each PA, respectively.

### 2.7. Statistical analysis

Mixing (M) of HV flow in the Fontan conduit was defined as follows: no mixing less than 0.1, poor mixing 0.1–0.3, mild mixing 0.3–0.5, moderate mixing 0.5–0.7, good mixing 0.7–0.9 and uniform mixing greater than 0.9. Bland–Altman plots and intraclass correlation (ICC) analysis were used to assess agreement between HFD methods. The mean absolute difference (±1.96 standard error of the mean) between methods was quantified to determine the absolute amount of which one HFD quantification method over- or underestimates the other method. All differences were reported in percentage points. Measurements were compared using a paired *t*-test. *M*_average_ between subsections was compared using a repeated measurement one-way ANOVA test with *post hoc* analysis (Tukey). A *p*-value <0.05 was considered statistically significant. Continuous data were presented as mean ± s.d. Data were analysed with SPSS 25.0 and Prism 8.0.

## 3. Results

Patient characteristics and MRI flow measurements are presented in table 1.

### 3.1. Mixing of inferior vena caval and hepatic venous flow

An evident streaming pattern of HV flow within the Fontan conduit was present resulting in non-uniform distribution of HV flow within the Fontan conduit, most evidently shown in the caudal part of the Fontan conduit (figure 1*a*,*b*; electronic supplementary material, video S1). This observation was also reflected by a significant lower cardiac cycle averaged mixing (*M*_average_) in the caudal (moderate mixing, mean 0.66 ± 0.13) compared to the cranial part of the Fontan conduit (good mixing, mean 0.79 ± 0.11, *p* < 0.001). *M*_average_ over the entire cross-section at both the caudal and cranial level of the Fontan conduit were significantly different from 0.9 (i.e. uniform mixing), *p* < 0.001 and *p* = 0.012, respectively. The mixing of HV and IVC blood flows within the caudal and cranial part of the Fontan conduit for a typical extracardiac conduit Fontan patient is shown in figure 1 and electronic supplementary material, video S1. The time-resolved mixing between HV and IVC flow in the four subsections at two different levels of the Fontan conduit is shown for a typical extracardiac Fontan patient (figure 1*d*,*e*).

When comparing *M*_average_ at the four subsections in the caudal cross-section, *M*_average_ at the right-posterior subsection was lowest, significantly lower compared to the right-anterior subsection (0.47 ± 0.34 versus 0.76 ± 0.20, respectively, *p* = 0.03). No significant differences between other caudal subsections (left-posterior 0.68 ± 0.29, left-anterior 0.65 ± 0.24) were observed. *M*_average_ was significantly different from 0.9 (i.e. uniform mixing) for all caudal subsections (all *p*-values <0.025). At the cranial level, no significant differences in *M*_average_ were found between the four subsections: right-anterior (0.85 ± 0.13), left-anterior (0.80 ± 0.18), right-posterior (0.75 ± 0.23) or left-posterior (0.73 ± 0.19). *M*_average_ was significantly different from 0.9 in the right- and left-posterior subsections (*p* = 0.02 and *p* = 0.004, respectively), indicating non-uniform mixing in these segments, but not in the right- and left-anterior subsections (*p* = 0.2 and *p* = 0.06, respectively).

### 3.2. Hepatic blood flow distribution analysis

**Figure 3.**
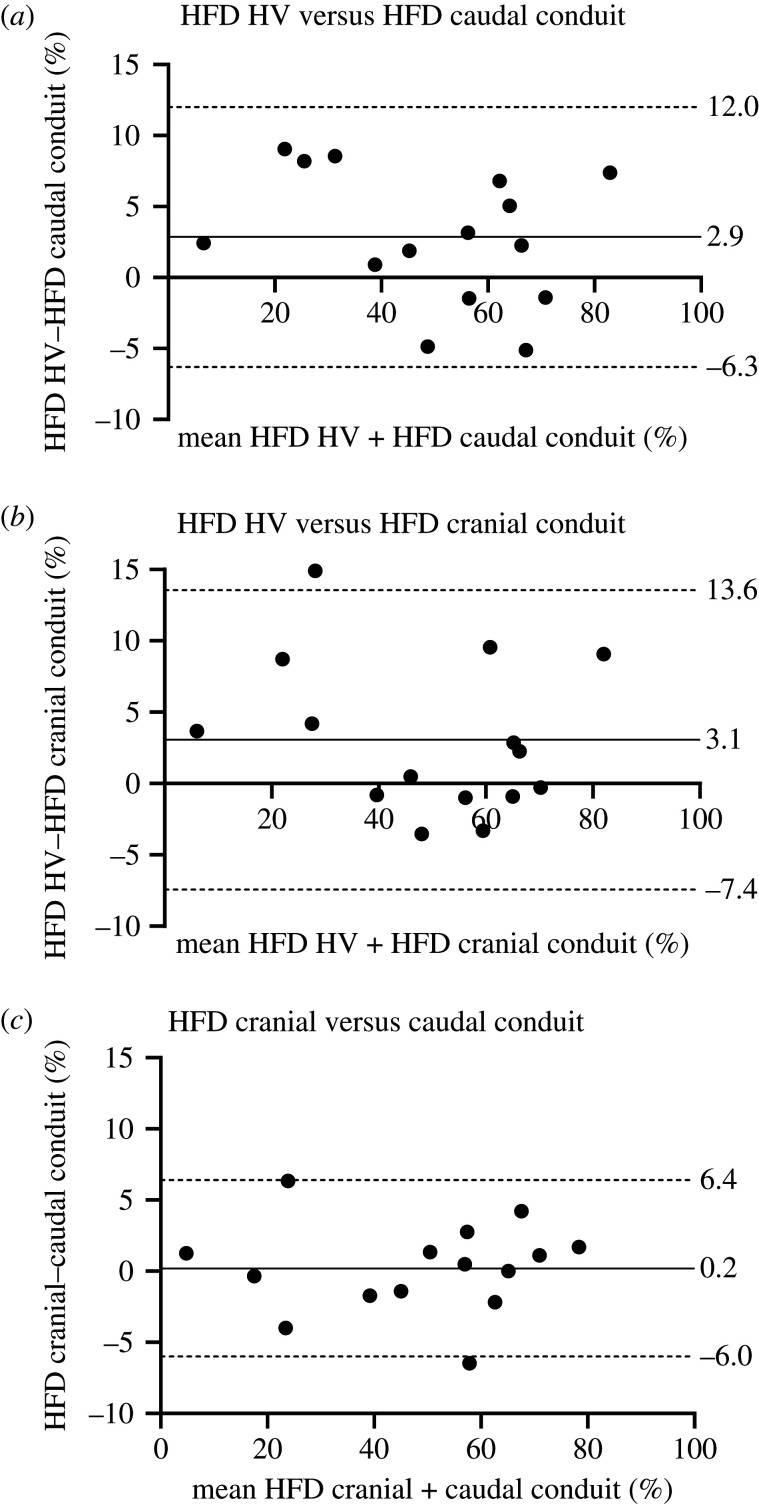
Bland–Altman plots comparing HFD measurements between the three different approaches. The mean difference + limits of agreement (±1.96 times the standard deviation) are shown in the plot.

The result of the HFD analysis and comparison between the three methods are presented in table 2 and figure 3. No significant differences were found between HFD_caudal conduit_ and HFD_cranial conduit_ (*p* = 0.80). However, when comparing the direct HFD_HV_ method with the conventional methods, significant differences were observed: HFD_HV_ (51.0 ± 20.6%) versus HFD_caudal conduit_ (*p* = 0.033) as well as HFD_HV_ versus HFD_cranial conduit_ (*p* = 0.044). Although statistically significant, differences between the conventional and direct HFD quantification methods were relatively small, with differences less than 5% in 8/15 and 11/15 using the HFD_caudal conduit_ and HFD_cranial conduit_ methods, respectively. However, in individual subjects, differences as high as 8.2–14.9% were observed in 4/15 patients.

**Table 2.** Comparisons between HFD quantification methods.

	paired *t*-test	Bland–Altman		intraclass correlation	mean absolute difference (%)
comparisons	*p* value	mean difference (%)	LoA (%)	ICC	(±1.96 SEM)
HFD_HV_ versus HFD_caudal tunnel_	0.033	2.9	−6.3–12.0	0.97	4.6 (3.1–6.0)
HFD_HV_ versus HFD_cranial tunnel_	0.044	3.1	−7.4–13.6	0.96	4.4 (2.2–6.5)
HFD_caudal tunnel_ versus HFD_cranial tunnel_	0.80	0.2	−6.0–6.4	0.99	2.4 (1.3–3.4)

HFD, hepatic flow distribution; SEM, standard error of the mean; LoA, limits of agreement, defined as the mean difference ±1.96 standard deviations.

No significant correlation was found for the absolute difference in HFD between the direct and both conventional methods and *M*_average_ at the caudal or cranial conduit, respectively (*r* = 0.32, *p* = 0.27 and *r* = −0.06, *p* = 0.82, respectively). The four patients with highest absolute differences between methods did not have a significantly different HV to IVC flow ratio (mean ratio 0.50 versus 0.54, *p* = 0.41), pulsatility (*Q*_max_−*Q*_min_/*Q*_mean_, where *Q* is the flowrate) of the IVC (mean 0.40 versus 0.41, *p* = 0.66) and conduit (mean 0.66 versus 0.53, *p* = 0.41) or HV to conduit percentage (mean 32% versus 35%, *p* = 0.75) compared to the 11 patients with smallest differences.

### 3.3. Comment

This study incorporates the hepatic veins into patient-specific CFD models of the TCPC in Fontan patients, in order to assess the mixing of HV and IVC flow and to allow for HFD quantification by tracking particles directly from the HVs. Main findings show that hepatic venous flow is not uniformly distributed within the Fontan conduit. This lack of uniform distribution was most evidently present at the caudal part of the Fontan conduit (just distal of the entry of the HVs into the IVC) with significantly better mixing of HV and IVC flows at the cranial part of the conduit. Tracking particles from the caudal or cranial part of the Fontan conduit did not result in significantly different HFD despite these different mixing characteristics. However, tracking particles directly from the HVs (HFD_HV_) was significantly different from both conventional HFD quantification methods (i.e. tracking Fontan conduit flow), although on a cohort level differences were small. On an individual basis, however, differences of 8.2–14.9% in HFD were observed which may be of clinical importance.

HFD quantification is an important metric in the evaluation of the TCPC in Fontan patients, since there is compelling evidence for a strong association between a lack of HV flow towards the lung and the formation of pulmonary arteriovenous malformations [2]. Therefore, the identification of an unbalanced HFD may indicate the need for intervention aiming to restore a more balanced HFD. Furthermore, when an intervention of the TCPC is considered, virtual surgery platforms offer the possibility to evaluate multiple different TCPC geometries [1,12,20]. Subsequently, the optimal geometry with minimal power loss while ensuring a balanced HFD can be selected [6,21,22]. The accuracy of the HFD quantification method itself, however, has not been studied.

The conventional approach for HFD quantification relies on the assumption that hepatic venous flow, theoretically taking up to approximately 38% of total Fontan conduit flow [23], is uniformly distributed within the Fontan conduit and thus can be used interchangeably. This study showed that this assumption is not valid, most evident at the level just above the entry of the hepatic veins into the IVC. The HV flow and IVC flow demonstrate two different flow streams with only moderate mixing during the cardiac cycle. This can be explained by the fact that blood flow in the Fontan conduit is in general laminar with only minimal pulsatility along the cardiac cycle preventing thorough mixing of both flows. The right-posterior part of the caudal Fontan conduit showed the lowest mixing of HV with IVC flow as the anatomy of the IVC and HVs likely resulted in less HV flow particularly in this segment. At the cranial side of the Fontan conduit, as both flows had more time to interact, significantly improved mixing was present but still non-uniform mixing was observed.

Therefore, using Fontan conduit flow as a surrogate of HV flow will introduce inaccuracies in HFD quantification. This study shows that on a cohort level the anticipated inaccuracy is small and of minimal clinical relevance. Therefore, the fact that HV flow is not uniformly distributed in the Fontan conduit does in general not seem to make a significant difference in HFD quantification, with 95% of mean absolute differences expected to be less than 6.1–6.5% making previously obtained results on a cohort level valid [3,20]. Furthermore, results also indicated that when the conventional HFD quantification is used, starting position of the particles (from the caudal or cranial conduit) did not result in significantly different HFD, despite the difference in mixing characteristics.

Although differences were small on a cohort level, it should be noted that on an individual basis, differences of 8.2–14.9% were observed in 4/15 patients which may have an impact on patient-specific clinical decision making. For example, in the patient with the largest difference, HFD was only 20.7% by tracking cranial conduit flow, while HFD was 35.6% when directly tracking HV flow. Although the minimal amount of HFD to prevent the formation of pulmonary arteriovenous malformations is not clear, a minimum HFD of 30% towards a lung has been suggested to be clinically acceptable [13,22,24]. For this patient, therefore, the conventional method would imply insufficient HFD, while direct HV flow tracking shows sufficient HV flow towards the LPA. Since virtual surgery platforms are used for pre-interventional planning on an individual basis, incorporating hepatic veins into the patient-specific CFD models might have an impact on at least some patients. As for the majority of patients the differences were small, it would be interesting to investigate if certain flow or geometric characteristics, which can show a wide variability at the level where the IVC and HVs join [25], can help identify the patients with the largest inaccuracies that would benefit most from direct HFD quantification. Because of the limited number of patients, this question could not be answered in this study. Larger numbers are needed in future studies to identify anatomical and/or flow characteristics to identify patients with important differences in direct and indirect HFD quantification.

### 3.4. Limitations

Although this study reveals important, novel insights into the flow dynamics of HV and IVC flow within the Fontan conduit and its influence on HFD quantification, some limitations are present. Although the sample size is relatively small, significant differences between methods could be detected with considerable differences on a patient-specific level. Furthermore, no direct hepatic venous flow measurements were acquired which were considered clinically unfeasible, by requiring multiple extra survey scans to plan 3–6 extra two-dimensional flow measurements in relatively small vessels. We assumed total hepatic venous flow to be distributed over the respective HVs by the ratio of their respective cross-sectional areas. Future studies should aim at obtaining direct measurements of HV flow in Fontan patients, for example, using four-dimensional flow MRI, to further increase the accuracy of HV flow modelling in these patients. In addition, ECG-gated, free-breathing two-dimensional PC-MRI was used as boundary conditions, which does not take respiration effects on hepatic flow into account. Previous studies have shown that hepatic venous flow can increase up to threefold during inspiration while having minimal effect on IVC flow [23], which may influence HV streaming patterns and subsequent HFD quantification. Also, predominantly extracardiac conduit Fontan patients were included in this study, and possible different flow characteristics in lateral tunnel patients may provide different results. Furthermore, a relatively coarse assessment of mixing was performed by dividing the cross-section of the Fontan conduit in only four parts. Therefore, mixing on a smaller scale was not taken into account.

## 4. Conclusion

In conclusion, hepatic venous flow is non-uniformly distributed within the Fontan conduit. Evident separate HV and IVC flow streaming patterns were present, most clearly just above the entry of the HVs into the IVC. On a cohort level, significant but small differences in HFD were observed when comparing direct (tracking hepatic venous flow) and conventional methods (tracking Fontan conduit flow). However, individual differences of 8.2–14.9% in HFD were observed in a subset of patients which may be of clinical importance by affecting accurate identification of patients with unbalanced HFD or by affecting optimal TCPC geometry selection when using virtual surgery platforms.

## Data accessibility

Available upon reasonable request.
